# In Silico Approaches to Polyherbal Synergy: Protocol for a Scoping Review

**DOI:** 10.2196/56646

**Published:** 2024-06-10

**Authors:** Anjana Chandhiruthil Sathyan, Pramod Yadav, Prashant Gupta, Arun Kumar Mahapathra, Ruknuddin Galib

**Affiliations:** 1 Department of Rasa Shastra and Bhaishajya Kalpana All India Institute of Ayurveda Delhi India; 2 Ayurinformatics Laboratory Department of Kaumarbhritya All India Institute of Ayurveda Delhi India

**Keywords:** polyherbal formulation, Ayurveda system, Ayurveda, Ayurvedic medicine, Ayurvedic treatment, herbal, herbal drug, pharmacodynamic, pharmacology, computer-aided drug design, in silico methodology, scoping review

## Abstract

**Background:**

According to the World Health Organization, more than 80% of the world’s population relies on traditional medicine. Traditional medicine is typically based on the use of single herbal drugs or polyherbal formulations (PHFs) to manage diseases. However, the probable mode of action of these formulations is not well studied or documented. Over the past few decades, computational methods have been used to study the molecular mechanism of phytochemicals in single herbal drugs. However, the in silico methods applied to study PHFs remain unclear.

**Objective:**

The aim of this protocol is to develop a search strategy for a scoping review to map the in silico approaches applied in understanding the activity of PHFs used as traditional medicines worldwide.

**Methods:**

The scoping review will be conducted based on the methodology developed by Arksey and O’Malley and the recommendations of the Joanna Briggs Institute (JBI). A set of predetermined keywords will be used to identify the relevant studies from five databases: PubMed, Embase, Science Direct, Web of Science, and Google Scholar. Two independent reviewers will conduct the search to yield a list of relevant studies based on the inclusion and exclusion criteria. Mendeley version 1.19.8 will be used to remove duplicate citations, and title and abstract screening will be performed with Rayyan software. The JBI System for the Unified Management, Assessment, and Review of Information tool will be used for data extraction. The scoping review will be reported based on the PRISMA-ScR (Preferred Reporting Items for Systematic Reviews and Meta-Analyses extension for Scoping Reviews) guidelines.

**Results:**

Based on the core areas of the scoping review, a 3-step search strategy was developed. The initial search produced 3865 studies. After applying filters, 875 studies were short-listed for further review. Keywords were further refined to yield more relevant studies on the topic.

**Conclusions:**

The findings are expected to determine the extent of the knowledge gap in the applications of computational methods in PHFs for any traditional medicine across the world. The study can provide answers to open research questions related to the phytochemical identification of PHFs, criteria for target identification, strategies applied for in silico studies, software used, and challenges in adopting in silico methods for understanding the mechanisms of action of PHFs. This study can thus provide a better understanding of the application and types of in silico methods for investigating PHFs.

**International Registered Report Identifier (IRRID):**

PRR1-10.2196/56646

## Introduction

Herbal medicine plays a significant role in treatment in various traditional medical systems worldwide [1-5]. Among such traditional medicine systems, Ayurveda has been a cornerstone of health care for a significant portion of the Indian population for centuries [6]. Despite its long-standing use, the scientific community’s acceptance of Ayurveda as an evidence-based health system remains tempered due to the lack of evidence-based data regarding the mode of action of Ayurvedic formulations.

Over the last 3 decades, substantial effort has been made to assess the effectiveness of Ayurvedic formulations. Traditional pharmacology tools, including in vivo and in vitro models, have been the most common means used for evaluating the preclinical efficacy of these formulations [7]. However, a persistent issue has been the lack of a detailed explanation regarding how these compounds work within the framework of modern science. This poses a challenge in establishing a robust scientific evidence base for Ayurveda. Thus, further efforts are needed to address the critical question of how Ayurvedic formulations work to justify their widespread use in health care.

There has been a notable shift toward the development and application of computational and in silico methods in pharmacological research [8]. These techniques have increasingly been used for formulating and testing various hypotheses related to a drug’s mode of action. In particular, in silico screening has emerged as a powerful tool for predicting a drug’s potential mechanism of action and its impact on specific target proteins of the human body. Thus, in silico studies that can identify lead compounds in polyherbal formulations (PHFs) and subsequently predict the molecular mechanism of action hold immense promise in the domain of Ayurvedic drug development.

To date, in silico approaches in understanding traditional medicine have mainly focused on single herbs. Although individual herbs contain hundreds of diverse phytochemicals, when combined to form PHFs, some of the original phytochemicals may be lost. In addition, the manufacturing process of a PHF may introduce new phytochemicals, resulting in a complex and dynamic mixture of metabolites [9]. This complexity necessitates a distinct approach in understanding the pharmacological properties of PHFs.

Although established methods are available for conducting in silico studies on individual herbs, little is known about strategies for in silico studies on PHFs. Therefore, the aim of this scoping review is to identify and chart the diverse in silico approaches that are currently being explored to understand the mechanism of action of PHFs, which can further help to identify the existing knowledge gaps in this area to target efforts for future research directions.

A preliminary search of MEDLINE, Cochrane Database of Systematic Reviews, and Joanna Briggs Institute (JBI) Evidence Synthesis database yielded no ongoing or completed scoping reviews on this specific topic. Apart from Ayurveda, other traditional medicine systems also depend on PHFs for the management of diseases. Considering the primary focus on PHFs, our search scope will extend to other traditional systems of medicine, ensuring a comprehensive understanding of the potential applications of in silico techniques in studying PHFs across different medical systems.

## Methods

### Study Design and Registration

This scoping review protocol was developed according to the framework proposed by Arksey and O’Malley [10] and complies with the recommendations of the JBI for elaborating scoping reviews [11]. A similar study from Brazil on in silico approaches in drug repurposing served as a guide in formulating this scoping review protocol [12]. This protocol has been registered with Open Science Framework [13] and is reported according to the Preferred Reporting Items for Systematic Reviews and Meta-Analyses extension for Scoping Reviews (PRISMA-ScR) guideline [14].

### Objectives and Search Strategy

The following questions were established to guide the review and literature search strategy: (1) How are the phytochemicals of PHFs identified? (2) What are the criteria for target identification? (3) Which in silico strategies are used in PHFs? (4) What is the methodology used in in silico studies of PHFs? (5) What are the challenges in adapting in silico studies in PHFs? (6) How are the results interpreted in the purview of the mode of action of PHFs? (7) How successful are these methods in proving the findings of in silico studies using other experimental methods?

A detailed 3-step search strategy will be developed by comparing the search terms and the related Medical Subject Heading (MeSH) terms. Using the developed search strategy in PubMed as a base, the search will further extend to the Embase, Science Direct, Web of Science, and Google Scholar databases to identify relevant articles on the topic. The search strategy will combine all identified keywords, MeSH terms, their relevant synonyms, and the Boolean operators “AND” and “OR.” The following three concept clusters were included: (1) polyherbal formulation, (2) in silico approach, and (3) mechanism of drug action.

### Study Selection

#### Inclusion Criteria

Peer-reviewed research articles published from January 2021 to December 2023 that incorporated in silico strategies in PHFs will be included. The rationale for this date range is to ensure that we capture the most current and relevant research findings related to in silico studies in PHFs, as the field of in silico research is continuously evolving. Studies published in any language will be included. Moreover, no limitations will be placed with respect to the system of medicine. In this regard, in silico studies related to PHFs used in any system of traditional medicine will be eligible for inclusion in the scoping review, including, but not limited to, Ayurveda.

#### Exclusion Criteria

Studies will be excluded if they meet the following criteria*:* in silico studies focusing solely on single drugs; studies in which only network pharmacology is employed (single drugs and PHFs); and reviews (mini, narrative, scoping, and systematic reviews), editorials, commentaries, letters, opinions, and grey literature.

### Study/Source of Evidence Selection

After completion of the search, all identified articles will be collected and organized using Mendeley software version 1.19.8. Any duplicate citations will be removed at this stage [15]. The titles and abstracts will then be subjected to screening by two or more independent reviewers using Rayyan software [16] in line with the predetermined inclusion criteria for the review.

Sources that are found to be potentially relevant will undergo a comprehensive review, and the citation details will be integrated into the JBI System for the Unified Management, Assessment, and Review of Information (SUMARI) for full-text assessment. Two or more independent reviewers will evaluate the selected citations against the preestablished inclusion criteria [17]. Any sources of evidence assessed at the full-text stage that do not meet the inclusion criteria will be clearly documented and the reasons for exclusion will be reported in the scoping review. Any disagreements among the reviewers at any stage of the selection process will be addressed through discussion or by involving additional reviewers for resolution.

The results of the search and the entire study inclusion process will be presented in full in the final scoping review. A PRISMA-ScR flow diagram will be included in the report to provide an overview of the selection process and outcomes.

### Data Extraction

Data will be extracted from articles included in the scoping review by two or more independent reviewers using the JBI SUMARI data extraction tool. This scoping review intends to yield a descriptive summary of the results.

The components listed in Textbox 1 will be considered while extracting the data. A data extraction pilot will be performed with 5 selected studies. The insights gained from the pilot study will be used to modify the draft data extraction tool as necessary during the process of extracting data from each included study. Any modifications will be detailed in the final scoping review. In the case of missing data or additional data, the authors of the papers will be contacted.

**Components of data to be extracted from the selected studies for the scoping review.**

- Title
- Date of publication
- Authors
- Country
- Study objectives
- Study design
- Name of the polyherbal formulation (PHF)
- Phytochemical analysis of the PHF
- In silico method implemented
- Limitations/challenges of the study
- Study outcome
- Proof of findings (supported by in vitro/in vivo experiments)

### Collating, Summarizing, and Reporting the Results

The data gleaned from the included studies will be presented in a descriptive format. This will involve a summary of the key findings, methodological approaches, and study characteristics. To facilitate understanding and enhance the clarity of the data collected, tables and graphs will be used. The results will be charted to provide an organized overview of the findings from the included studies. This charting process will categorize the data according to the components of data extraction listed in Textbox 1. Findings will be dissected and discussed explicitly in relation to the predefined questions of the scoping review. The scoping review will adhere to the PRISMA-ScR guidelines to ensure a rigorous and transparent reporting process.

## Results

An initial search was performed on the PubMed database using the keywords and strategy outlined in Table 1. The initial search yielded 3865 studies. After applying filters such as full-text availability, articles published in English, and those published between January 2021 and December 2023, 875 studies were obtained. As the study progressed, the search strategy was changed. The Boolean term “NOT” was removed and additional keywords were included.

**Table 1:** Initial search strategy for the scoping review performed in PubMed in December 2023.

Step	Search strategy	Yielded studies
1	((((((polyherbal formulation) OR (Traditional formulations)) OR (Traditional Medicine)) AND (in silico)) OR (molecular docking)) OR (Molecular dynamic)) AND (mechanism Drug Action))	3865
2	Filters: text availability: Full Text	1867
3	Filters: Published between January 2021 to present date	878
4	Filters: Language: English	875

## Discussion

The findings of this scoping review are expected to address the specific review questions and identify knowledge gaps related to the methods used for the phytochemical identification of PHFs, determine the criteria for target selection, and propose evidence-backed methods for conducting in silico studies on PHFs.

This scoping review will be the first of its kind in providing a comprehensive understanding of the potential applications of in silico techniques in studying PHFs across different traditional medicine systems. The findings will also be interpreted in a manner that can facilitate evaluating the effectiveness of these methods for gaining a better understanding of the mechanism of drug action.

In silico approaches have proven to be significant in identifying novel drug candidates and repurposing existing drugs [12,18,19]. These studies have also been instrumental in shedding light on the likely mechanisms of action of single herbal drugs [20]. However, the in silico strategies used to study PHFs are not well understood. Several methods appear to be used to conduct in silico studies on PHFs [21,22]. This scoping review can thus help to identify the most effective in silico methods in this context and can further help to guide the design of future in silico studies on various types of PHFs used in traditional medicine.

The scoping review is limited by its reliance on in silico studies, which can offer valuable preliminary insights but may not fully capture the complex interactions and bioavailability of compounds within PHFs when administered in vivo. Additionally, this review might encounter limitations due to the variability of methodologies and the quality of reporting across in silico studies, potentially affecting the consistency and comparability of findings related to the efficacy and safety of PHFs.

**Figure 1:**
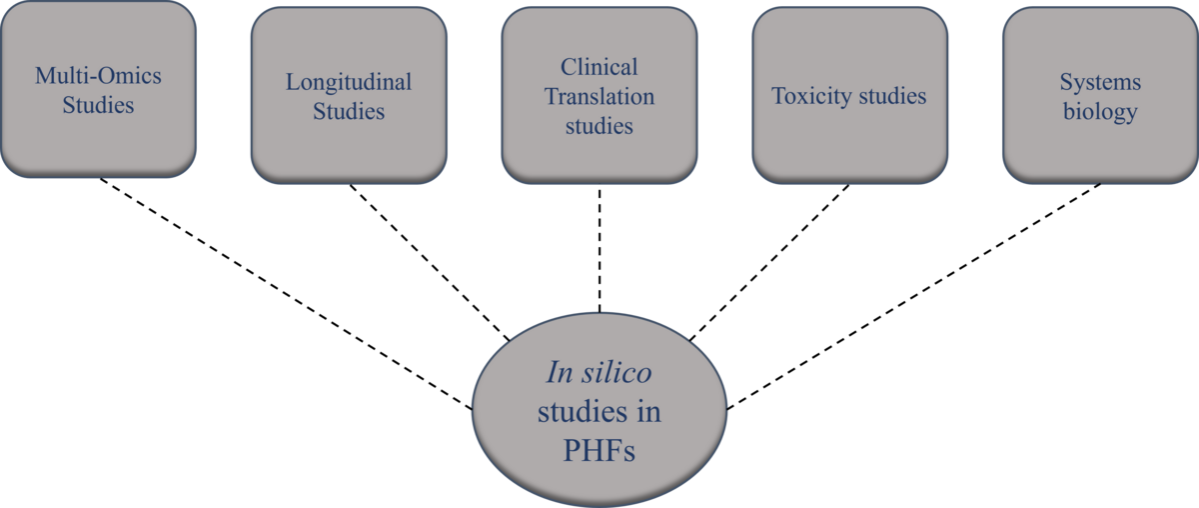
Future perspectives of scoping reviews of in silico studies related to polyherbal formulations (PHFs).

The future scope and perspectives of this field that can be informed by this scoping review are depicted in Figure 1. The results of in silico studies can be further validated through preclinical and clinical studies. Multiomics strategies such as genomics and metabolomics can also be applied. The review seeks to provide an overview of the progress made in employing in silico techniques to decipher the mechanisms underlying the efficacy of PHFs, thereby contributing to our understanding of this critical aspect of traditional medicine.

## Abbreviations

JBI: Joanna Briggs Institute
MeSH: Medical Subject Heading
PHF: polyherbal formulation
PRISMA-ScR: Preferred Reporting Items for Systematic Reviews and Meta-Analyses extension for Scoping Reviews
SUMARI: System for the Unified Management, Assessment, and Review of Information
